# Supplementary material for: Unveiling the Potentiality of Shikonin Derivatives Inhibiting SARS-CoV-2 Main Protease by Molecular Dynamic Simulation Studies
Source: Int J Mol Sci. 2023 Feb 4;24(4):3100. doi: 10.3390/ijms24043100 (PMC9963214; doi:10.3390/ijms24043100)
Supplement: Supplementary file 1 [file ijms-24-03100-s001.zip › ijms-2149144-Supplementary.pdf]

Supplementary Data

Unveiling the potentiality of Shikonin Derivatives inhibiting SARS-CoV-2 main protease

Table S1: List of Shikonin derivatives according to different SAR modifications.

Compound name	IUPAC Name	Structure	Binding affinity (kcal/mol)
1,4-napthoquinone	Naphthalene-1,4-dione	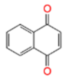	-3.853
Acetylshikonin	[(1*R*)-1-(5,8-dihydroxy-1,4-dioxonaphthalen-2-yl)-4-methylpent-3-enyl] acetate	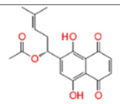	-5.838
Isobutyrylshikonin	[1-(5,8-dihydroxy-1,4-dioxonaphthalen-2-yl)-4-methylpent-3-enyl]2-methylpropanoate	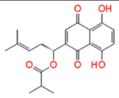	-5.957
Angelylshikonin	[4-methyl-1-(1,4,5,8-tetrahydroxy-2-naphthyl)penta-1,3-dienyl] 2-methylbut-2-enoate	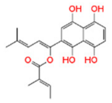	-5.974
3,4-methylenedioxyphenyl acetyl shikonin	1-(1,3-benzodioxol-5-yl)ethanone	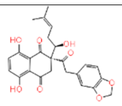	-4.707
4-chlorophenylacetyl shikonin	3-[2-(4-chlorophenyl)acetyl]-5,8-dihydroxy-3-[(1*R*)-1-hydroxy-4-methylpent-3-enyl]-2*H*-naphthalene-1,4-dione	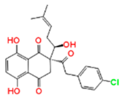	-5.747
Acryloyl shikonin	5,8-dihydroxy-2-[(1R)-1-hydroxy-4-methyl-pent-3-enyl]-2-prop-2-enoyl-tetralin-1,4-dione;ethane	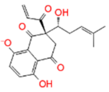	-5.733
Alpha-methylbutyryl shikonin	5,8-dihydroxy-3-[(1*R*)-1-hydroxy-4-methylpent-3-enyl]-3-(2-methylbutanoyl)-2*H*-naphthalene-1,4-dione	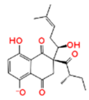	-3.829
Alpha-methyl-n-butyl shikonin	3-butyl-5,8-dihydroxy-3-[(1*R*)-1-hydroxy-4-methylpent-3-enyl]-2-methyl-2*H*-naphthalene-1,4-dione	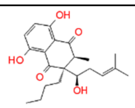	-5.986

Arnebinone	8-ethenyl-2,3-dimethoxy-8-methyl-5-prop-1-en-2-yl-6,7-dihydro-5*H*-naphthalene-1,4-dione	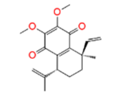	-4.342,
Beta-beta-dimethylacryl shikonin	[(1*R*)-1-(5,8-dihydroxy-1,4-dioxonaphthalen-2-yl)-4-methylpent-3-enyl] 3-methylbut-2-enoate	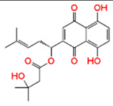	-5.641
Beta-Hydroxyisovaleryl shikonin	[(1*R*)-1-(5,8-dihydroxy-1,4-dioxonaphthalen-2-yl)-4-methylpent-3-enyl] 3-hydroxy-3-methylbutanoate	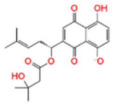	-5.628
Deoxyshikonin	5,8-dihydroxy-2-(4-methylpent-3-enyl)naphthalene-1,4-dione	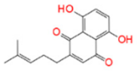	-6.179
Lithospermidin-B	[(*E*)-1-(1,4-dihydroxy-5,8-dioxonaphthalen-2-yl)-4-hydroxy-4-methylpent-2-enyl] 3-methylbutanoate	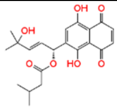	-6.005
Propionyl shikonin	[(1*R*)-1-(5,8-dihydroxy-1,4-dioxonaphthalen-2-yl)-4-methylpent-3-enyl] propanoate	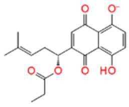	-3.153
Shikonin	5,8-dihydroxy-2-[(1*R*)-1-hydroxy-4-methylpent-3-enyl]naphthalene-1,4-dione	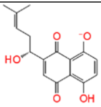	-2.954
Shikonin acetate	5,8-dihydroxy-2-[(1*R*)-1-hydroxy-4-methylpent-3-enyl]-1,4-dioxo-3*H*-naphthalen-2-yl] acetate	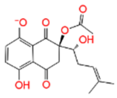	-5.823
Shikonin glucoside	5,8-dihydroxy-2-[4-methyl-1-[3,4,5-trihydroxy-6-(hydroxymethyl)oxan-2-yl]oxypent-3-enyl]naphthalene-1,4-dione	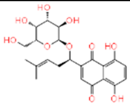	-8.079
Shikonin leucoacetate	[4,5,8-triacetyloxy-6-(1-acetyloxy-4-methylpent-3-enyl)naphthalen-1-yl] acetate	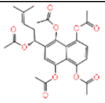	-4.230
Teracrylshikonin	[(1*R*)-1-(5,8-dihydroxy-1,4-dioxonaphthalen-2-yl)-4-methylpent-3-enyl] 3,4-dimethylpent-3-enoate	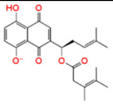	-5.337

Table S2: Docking and binding free energy result of the selected compounds.

**Compound Name**	**Docking score (kcal mol^{-1})**	**MM-GBSA (kcal mol^{-1})**	**MM-GBSA (kcal mol^{-1})**
Shikonin	-6.029	-26.96	-35.58
Shikonin Glucoside	-8.079	-53.026	-22.69
Lithospermidin-B	-6.094	-44.299	-36.52
Alpha methyl butyl shikonin	-5.631	-45.145	-35.33
Beta-hydroxyisovaleryl shikonin	-6.822	-45.145	-37.95

Table S3: Vital ADMET parameters

**Attributes**	**Values**
Molecular weight (MW)	<500
Hydrogen bond donors (HBD)	≤ 5
Hydrogen bond acceptors (HBA)	≤ 10
Predicted brain/blood partition coefficient (QPlog BB)	-3.0 to 1.2
Predicted IC50 value for blockage of HERG K+ channels (QPlog herg)	< –5
Human oral absorption (HOA)	3=higher, 2= medium, 1=lower
Predicted apparent MDCK cell permeability in nm/sec (QPlog mdck)	<25 poor,<br>>500 great

Table S4: ADMET results of selected compounds

ADMET properties	Shikonin Glucoside	Beta-hydroxyisovaleryl shikonin	Lithospermidin-B	Alpha-methyl-n-butyl shikonin	Shikonion
MW	450.441	390.432	388.416	360.449	288.299
HBD	4	1	1	0	0
HBA	13.7	6.95	5.25	4.2	4.2
QPlogPo/w	-0.341	2.714	3.08	3.948	2.123
QPlogS	-3.588	-4.841	-5.211	-5.249	-3.409
QPPCaco	11.541	293.637	66.408	546.005	120.678

QPlogBB	-3.293	-1.54	-2.36	-1.205	-1.68
HOA	1	3	3	3	3
SASA	711.333	633.394	702.049	624.029	547.115
FOSA	262.462	369.66	356.07	381.291	203.637
QPpolrz	39.639	35.096	39.378	35.05	28.791
QPlogPC16	14.417	11.151	12.572	10.606	9.283
QPlogPoct	26.381	16.618	18.175	13.593	11.917
CIQPlogS	-3.588	-4.841	-5.211	-5.249	-3.847
QPlogHERG	-5.251	-4.51	-5.306	-4.491	-4.706
QPPMDCK	3.98	131.558	26.382	257.212	50.315
QPlogKp	-5.577	-3.3168	-4.4469	-2.714	-4.051
QPlogKhsa	-0.84	0.012	0.407	0.444	-0.044
#metab	10	9	5	7	6

Table S5: Binding site analysis of Shikonin and selected top hits.

<table>
<tr><th>Compound Name</th><th>Hydrogen bonds (Distance in Å)</th><th>Hydrophobic bonds (Distance in Å)</th><th>π stacking</th></tr>
<tr><td rowspan="5">Beta-hydroxyisovaleryl shikonin</td><td>His^{164} (1.91)</td><td rowspan="3">Met^{165}(3.88)</td><td rowspan="5">His^{41}(4.14)</td></tr>
<tr><td>Glu^{166} (2.26)</td></tr>
<tr><td>Glu ^{166} (2.99)</td></tr>
<tr><td>Glu ^{166} (1.98)</td><td rowspan="2">Gln^{189}(3.87)</td></tr>
<tr><td>Asp^{187} (3.26)</td></tr>
<tr><td rowspan="3">Shikonin</td><td rowspan="3">Arg^{188}(3.04)</td><td>Met^{165}(3.78)</td><td rowspan="3">His^{41} (3.94)</td></tr>
<tr><td>Glu ^{166}(3.71)</td></tr>
<tr><td>Asp^{187}(3.70)</td></tr>
<tr><td rowspan="5">Alpha-methyl-n-butyl_shikonin</td><td rowspan="2">Arg ^{188}(3.42)</td><td>His^{41}(3.30)</td><td rowspan="5"></td></tr>
<tr><td>Met^{49}(3.82)</td></tr>
<tr><td rowspan="3">Arg ^{188}(2.59)</td><td>Leu^{167}(3.98)</td></tr>
<tr><td>Gln^{189}(3.71)</td></tr>
<tr><td>Gln^{192}(3.78)</td></tr>
<tr><td rowspan="3">Lithospermidin-B</td><td>Thr^{190}(3.19)</td><td>His^{41}(3.17)</td><td rowspan="3"></td></tr>
<tr><td>Thr^{190}(2.25)</td><td>Met^{49}(3.84)</td></tr>
<tr><td>Gln ^{192}(1.83)</td><td>Leu^{167}(3.78)</td></tr>
<tr><td rowspan="5">Shikonin_glucoside</td><td>Glu^{166}(2.06)</td><td>His^{41}(3.24)</td><td rowspan="5">His^{41}(3.48)</td></tr>
<tr><td>Glu ^{166}(2.16)</td><td>Met^{49}(3.47)</td></tr>
<tr><td>Thr^{190}(2.07)</td><td>Met^{165}(3.95)</td></tr>
<tr><td>Thr^{190}(3.16)</td><td rowspan="2">Gln^{189}(3.45)</td></tr>
<tr><td>Gln^{192}(2.24)</td></tr>
</table>

A.

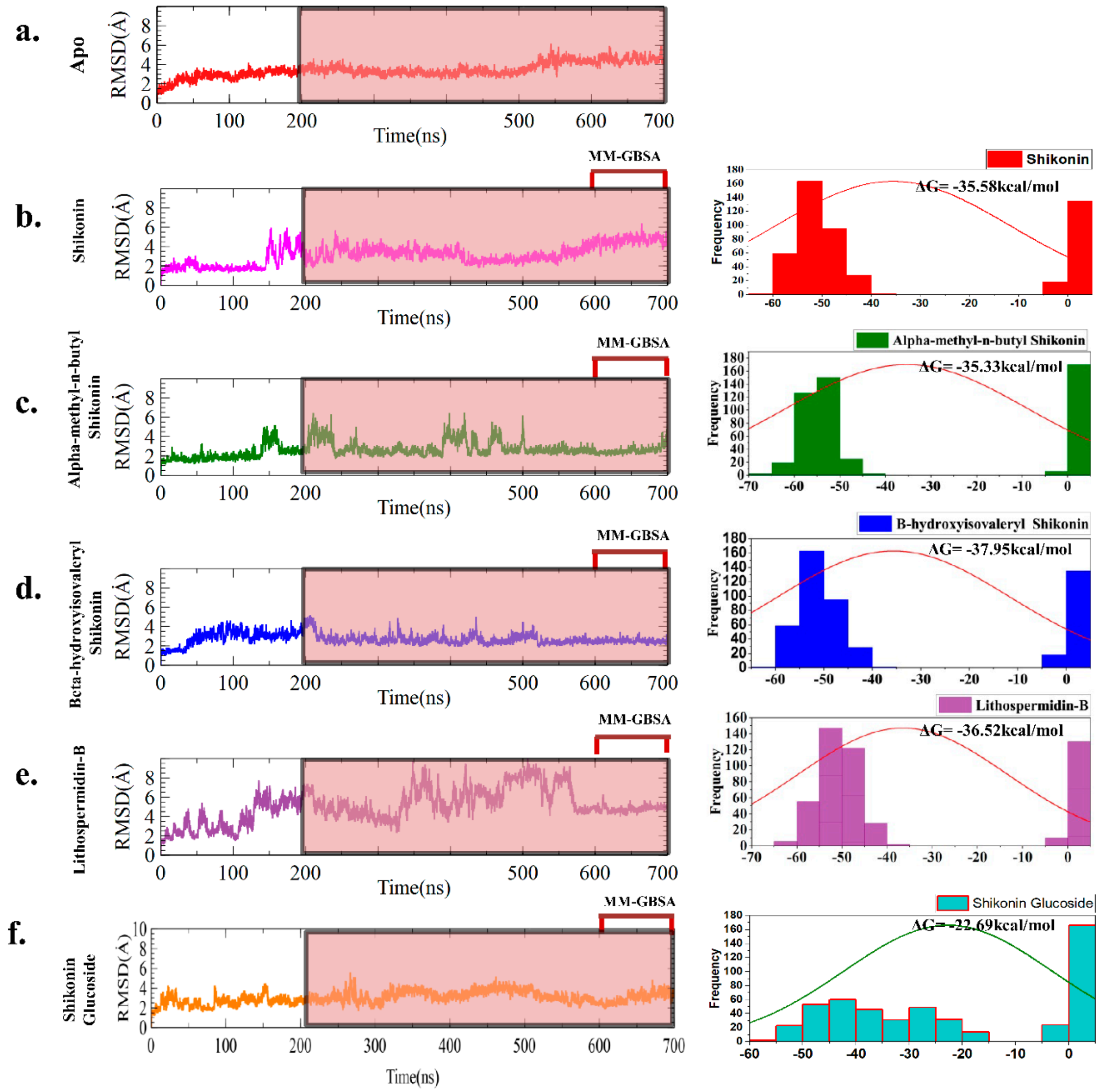

a.
Apo
RMSD(Å)
Time(ns)
b.
Shikonin
MM-GBSA
Shikonin
ΔG= -35.58kcal/mol
Frequency
c.
Alpha-methyl-n-butyl Shikonin
Alpha-methyl-n-butyl Shikonin
ΔG= -35.33kcal/mol
d.
Beta-hydroxyisovaleryl Shikonin
B-hydroxyisovaleryl Shikonin
ΔG= -37.95kcal/mol
e.
Lithospermidin-B
Lithospermidin-B
ΔG= -36.52kcal/mol
f.
Shikonin Glucoside
Shikonin Glucoside
ΔG= -22.69kcal/mol

B.

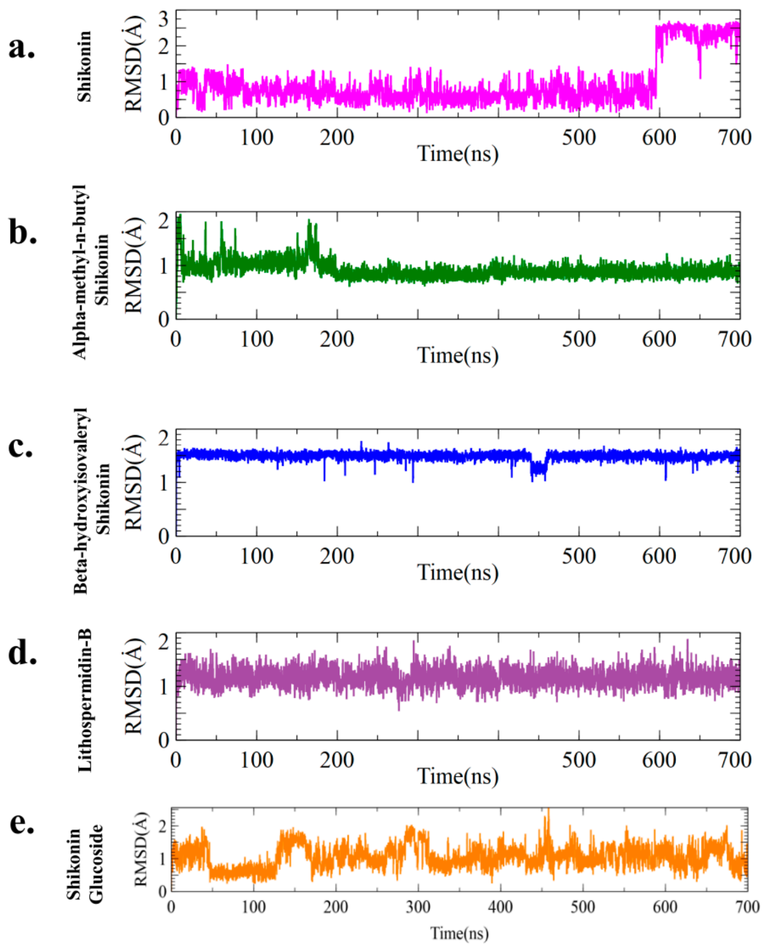

Figure S1: Time evolution of root-mean-square deviations (RMSDs). **A)** Protein RMSD for a) Apo, b) Shikonin, c) Alpha-methyl-n-butyl Shikonin., d) Beta-hydroxyisovaleryl Shikonin, e) Lithospermidin-B, f) Shikonin Glucoside **B)** Ligand RMSD for a) Shikonin, b) Alpha-methyl-n-butyl Shikonin., c) Beta-hydroxyisovaleryl Shikonin, d) Lithospermidin-B, e) Shikonin Glucoside.
